# Molecular investigation of the colicinogenic *Escherichia coli* strains that are capable of inhibiting *E. coli* O157:H7 in vitro

**DOI:** 10.1186/s12917-018-1771-y

**Published:** 2019-01-07

**Authors:** Nasrin Askari, Reza Ghanbarpour

**Affiliations:** 1 Department of Pathobiology, Faculty of Veterinary Medicine, Shahid Bahonar University of Kerman, Kerman, Iran; 2 Molecular Microbiology Research Group, Faculty of Veterinary Medicine, Shahid Bahonar University of Kerman, Kerman, Iran

**Keywords:** *Escherichia coli O157; H7*, Colicin, Sheep, Virulence genes, Phylogenetic groups, Probiotics

## Abstract

**Background:**

*Escherichia coli* O157:H7 is a highly virulent human pathogen with severe consequences following infection, which claims many lives worldwide. A suggested method for controlling this bacterium is the competitive elimination through using probiotic bacteria that prevent its colonization. Some nonpathogenic *E. coli* strains that produce antibacterial colicins are among these probiotic bacteria. We aimed to isolate and characterize the colicinogenic *E. coli* strains from diarrheic and healthy sheep that inhibit *E. coli* O157:H7, which could be used as possible probiotic sources. A total of 292 *E. coli* isolates (146 from each diarrheic and healthy sheep) were screened for the presence of colicin and virulence genes. The phylogenetic group/subgroup determination was performed by PCR. In vitro evaluation of inhibitory effect of colicinogenic isolates on *E. coli* O157:H7 was done phenotypically.

**Results:**

The frequency of diarrhea associated colicinogenic *E. coli* isolates was significantly higher than those isolated from healthy sheep. An association between ETEC and the genes coding for colicin-V & colicin-Iab in diarrheic *E. coli* isolates was observed. Moreover, there was an association between *ipaH* and Colicin-V encoding genes. Furthermore, *E. coli* isolates showing bacteriocinogeny while possessing no virulence genes had a frequency of 97.67 and 11.94% in healthy and diarrheic isolates, respectively. Of these strains, five isolates (3.42%) from diarrheic and twenty-five isolates (17.12%) from healthy sheep inhibited O157:H7 strain. Additionally, colicin E1 and colicin Iab genes were more prevalent in B1 phylogroup.

**Conclusions:**

These results signified that healthy sheep could be considered as a potential source for anti-O175:H7 bacterial isolates.

## Background

*E. coli* O157: H7 is well known as a severely virulent foodborne bacterial pathogen that claims many lives annually worldwide [1]. The clinical symptoms of *E. coli* O157:H7 vary wildly from mild diarrhea to hemolytic uremic syndrome (HUS) leading to kidney failure. The severity of these symptoms depends on the immune status of the patient and also the infection dose of the bacteria [2, 3]. It has been shown that the main reservoir of *E. coli* O157:H7 are ruminants [4].

However, one proposed approach for the control of *E. coli* O157:H7 is through the competitive elimination, which is described as the use of other probiotic bacteria to prevent the growth and colonization of the pathogenic bacterium [5]. The reduction of *E. coli* O157:H7 could be achieved through the use of other nonpathogenic *E. coli* strains that produce colicins [6]. Colicins are antimicrobial proteins synthesized by *E. coli* for inhibiting other *E. coli* strains as well as other closely related bacteria [7]. These proteins have evolved to provide the carrying bacterium an advantage when competing with the closely related bacteria [8]. The ability to produce colicins is common in the *Enterobacteriacea* family, and studies have revealed that approximately 30% of *E. coli* isolates are capable of producing at least one type of colicin [9].

Host animal species can effect on the type of produced colicin and bovine isolates would be expected to show low rate of resistance against to non-cattle Colicinogenic *E. coli* [5]. Zhao et al., [10] developed the first competitive- exclusion system against *E. coli* O157:H7 by isolating colicinogenic *E. coli* isolates from cattle, but little information was provided about the types of colicins produced by these isolates and the extent of resistance among naturally present *E. coli* O157:H7 isolates [11].

For determination of the pathogenic or non-pathogenic nature of *E. coli* isolates, assessment of the evolutionary origins via phylogenetic analysis has been helpful [12]. Based on the phylogenetic studies, *E. coli* isolates can be allocated to one of the four main phylogenetic groups A, B1, B2 and D, which can fall into seven subgroups [13].

Previously published studies have only provided partial insight into the association between colicin production and virulence factors, as they were mainly focused upon uropathogenic *Escherichia coli* (UPEC) isolates and differed in the number of detected colicin and virulence genes [14]. The present project was designed to genotypic analysis of colicinogenic *E. coli* isolates from diarrheic and healthy sheep that inhibit serotype O157:H7. Additionally, this research was set to characterized phylogenetic groups/subgroups of the isolates.

The work presented here is a continuation of our previous study. 146 of the diarrheic strains that are used in the current study were also used in a previous study where their virulence genes profiles were studied [15]. Here we aimed to further expand our investigations through analyzing these strains in terms of determination of colicin genes, phylogrouping and the inhibitory effect of them on serotype O157:H7 and comparing them with strains from healthy once.

## Methods

### Sample collection and isolation of *E. coli* strains

This section of the study was performed from March to Oct 2015. 167 fecal samples from healthy sheep were collected in Kerman province, Iran. Each sample was belonged to one animal which were between 1 to 12 weeks old. Swab samples were placed directly in tubes containing the Amies medium (Becton Dickinson, BBL, and USA) and sent to the laboratory for immediate processing. Samples were streaked on MacConkey agar (Merck, Germany) and incubated at 37 °C for 24 h. Colonies showing *E. coli* features were sent to Gram staining and were confirmed to be *E. coli* by using the biochemical API 20E identification system (BioMérieux, Marcy l’Etoile, France). One confirmed isolate was chosen from each plate. Finally, 146 *E.coli* strains from healthy sheep were stored in Luria- Bertani broth (Invitrogen, Paisley, Scotland) with 30% sterile glycerol at − 80 °C and as the above-mentioned, 146 of the diarrheic strains that are used in the current study were also used in a previous study [15]. Therefore, the assays were done on 292 isolates (146 from each diarrheic and healthy sheep).

Ten *E. coli* strains were used as positive controls, including H10407 (*LT-I +, ST-I+*); 1404 (*cdtIII +, cnf2 +, f17A+*); 510 (*F5 +, F41+*); 28C (*cdtIV +, cnf1+*); 31A (*f17c-A +*); Sakaï (*stx*_*1*_ *+, stx2+, eaeA+*); 85b (*ipaH+*); 25KH9 (*f17a-A+*), S5 (*f17b-A+*); and ECOR62 (*chuA +, yjaA +* and *Tspe4 C2+*). The laboratory nonpathogenic *E. coli* MG1655 was used as a negative control. All the above-mentioned reference strains had been taken from the bacterial collection section of the Microbiology Department of Ecole Nationale Vétérinaire Toulouse, Franc*e. coli*cin reference strains were provided from the Pasture Institute of Iran.

### Molecular detection of the virulence genes and phylogenetic groups

DNA extraction of the overnight *E. coli* cultures (current isolates and the reference isolates) was performed through the boiling method [15]. The virulence genes had been amplified and analyzed as described in details before. A complete list of the primers used for this virulence genotyping is published previously [15]. Additionally, the absence or presence of *yjaA, chuA*, and TSPE4.C2 sequences [13] in *E. coli* isolates (see Table 1), which were determined by multiplex PCR, was used to identify the phylogenetic groups.

**Table 1:** Specific primers used for PCR amplifications of genes used for phylotyping

Gene	Primer Sequence (5′-3′)	Band Size (bp)	Tm (°C)
*chuA*	GAC GAA CCA ACG GTC AGG AT TCG CCA GTA CCA AAG ACA	279	55
*yajA*	TGA AGT GTC AGG AGA CGC TG ATG GAG AAT GCG TTC CTC AAC	211	55
*TSP*	GAG TAA TGT CGG GGC ATT CA CGC GCC AAC AAA GTA TTA CG	152	55

### Molecular detection of the colicin genes

All *E. coli* isolates were examined by several PCR protocols for the presence of the colicin encoding genes including colicin-Iab, V, ANS4, E1, Mix E, 5, 10, K and colicin YU [16] PCR Amplification was performed in 25 μL volumes containing: 2.5 μL of 10× PCR buffer, 3 μL prepared DNA, 2 mM MgCl2, 0.3 M of each oligonucleotide primer, 0.2 mM dNTP mix, 1 U *Taq* DNA polymerase (Cinnagen, Iran), and PCR grade water up to 25 μL. The PCR conditions included 1 cycle of denaturation at 94 °C for 2 min, annealing at the Tm specific for each primer (see Table 2) for 1 min, and elongation for 1 min at 72 °C; subsequently 35 cycles of denaturation at 94 °C for 1 min and then a final 5 min at 72 °C hold temperature. The PCR products were electrophoresed on 1.5% agarose gel for 90 min at 85 v. The DNA bands were visualized through staining of gels with DNA Fluorescent Loading Dye (Sigma, Germany).

**Table 2:** PCR primer used for detection of Colicin genes

Name of Colicin	Primer Sequences (5′ to 3′)	Band Size (bp)	Tm (°C)
*Colicins A, N, S4*	CGT AGC TAT AAT GAA GCA ATG GCT TCA ACC TCC AAC AGG AGA GGT CCC CAG TT	225	57
*Colicin V*	CAC GCC CTG AAG CAC CAC CA CCG TTT TCC AAG CGG ACC CC	400	68
*Colicins Ιa, Ιb*	GCA CAA CAG GCC CGT CTG CTC CAC CTT CCA CAT CCT CTG TCA CC	385	68
*Colicins E2, E3, E4, E5,E6, E7, E8, E9*	CGA CAG GCT AAA GCT GTT CAG GT TGC AGC AGC ATC AAA TGC AGC CT	219	60
*Colicins U, Y*	GTG AAC GGA CAG AAA CCC GCC CAA TCT GTC TGA CAG CCT CTC CC	243	68
*Colicin 5, 10, K*	AAA GCT GAA CTG GCG AAG GC CAA CTC ATC ATC CCC TAT GTA AGA AG	803	60
*Colicin E1*	ACG GGA GTG GCT CTG GCG G CTC TTT ACG TCG TTG TTC TGC TTC CTG	389	68

### Evaluation of the inhibitory activity of *E. coli* strains

The sensitivity of the pathogenic *E. coli* O157:H7 Sakai (EHEC) to the colicins was examined here. The *E. coli* O157:H7 colonies and the colicin positive strains, which have been identified in the previous steps, were inoculated into Lysogeny Broth (LB; Merck, Germany) and were grown overnight (12 to 16 h). The overnight grown strain of *E. coli* O157:H7 was streaked and spread thoroughly on the surface of LB agar plates containing 0.025 μg of Mitomycin C (Sigma, USA) and were then allowed to dry. Mitomycin C’s role here was to induce the colicins production in the isolated strains. Fresh overnight cultures (7 μL from each colicinogenic isolates were spotted on the same LB plates that had been in the previous step streaked with *E. coli* O157:H7. These bacterial test plates were afterwards stored at 37 °C for 12 to 16 h. Finally, the clearance zones around the spotted *E. coli* isolates were measured [16]. The clear zone indicates no growth of *E. coli* O157:H7.

### Statistical analysis

SPSS software (version 17. SPSS Inc., USA) was used for the data analysis. *P* value was measured using Chi-square and Fisher’s exact tests to find any significant relationship. *P* values less than 0.05 were considered statistically meaningful.

## Results

PCR assay showed that sixty-seven *E. coli* isolates (45.89%) from diarrheic samples contained colicin encoding genes. Twenty-five (17.12%) isolates of them harbored colicin Iab encoding genes and most of these isolates (15.06%) contain the virulence genes related to ETEC pathotype. Eighteen (12.32%) isolates possessed colicin E1 encoding genes and were widely distributed among STEC (9.58%) pathotype. Fifteen *E. coli* isolates (10.27%) were positive for colicin V encoding genes and were distributed among ETEC (6.16%) and EIEC (0.68) pathotypes, respectively. Colicin-Iab/ E1/ V were the most prevalent colicin encoding genes and colicin Mix E and colicin 5, 10, k were not detected in any of the diarrheic *E. coli* isolates.

**Fig. 1:**
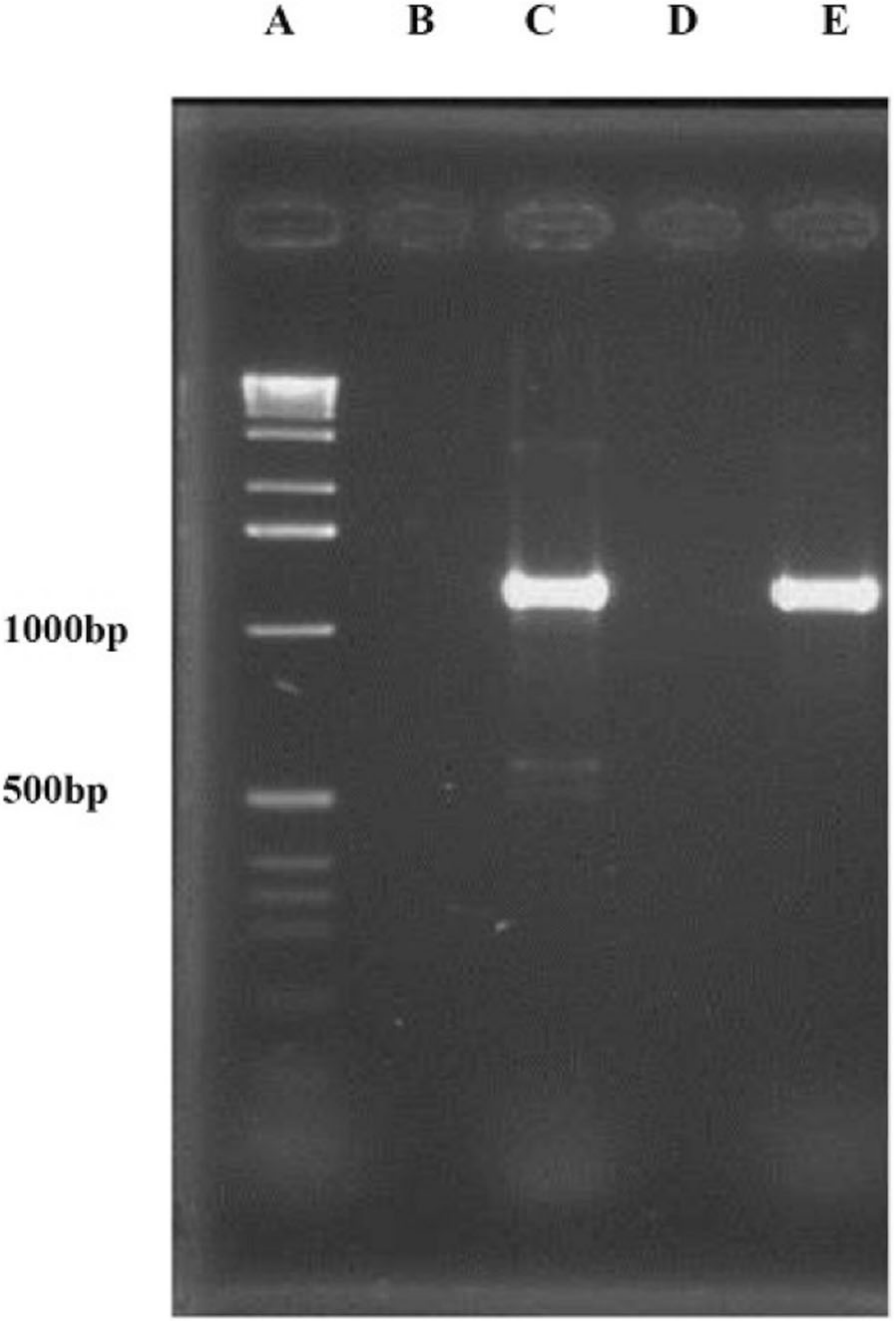
Molecular detection of NTEC strains. A) DNA Ladder, B) Negative control, C) Positive control (*E. coli* S5), D) Negative isolate for *cnf*1 gene (NTEC), and E) Positive isolate for *cnf*1 gene

**Fig. 2:**
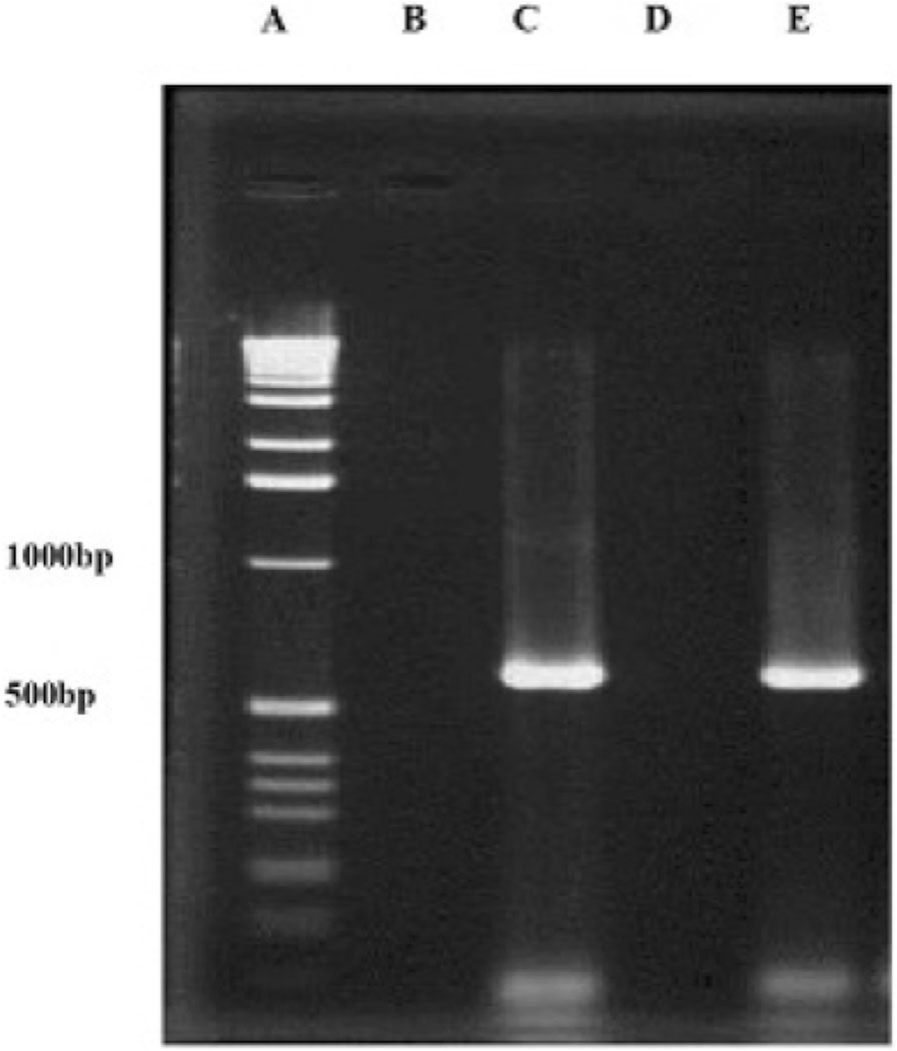
EIEC strain detection by PCR. A) DNA Ladder, B) Negative control, C) Positive control (*E. coli* 85b), D) Negative isolate for *ipaH* gene, and E: Positive isolate for *ipaH* gene (EIEC)

**Fig. 3:**
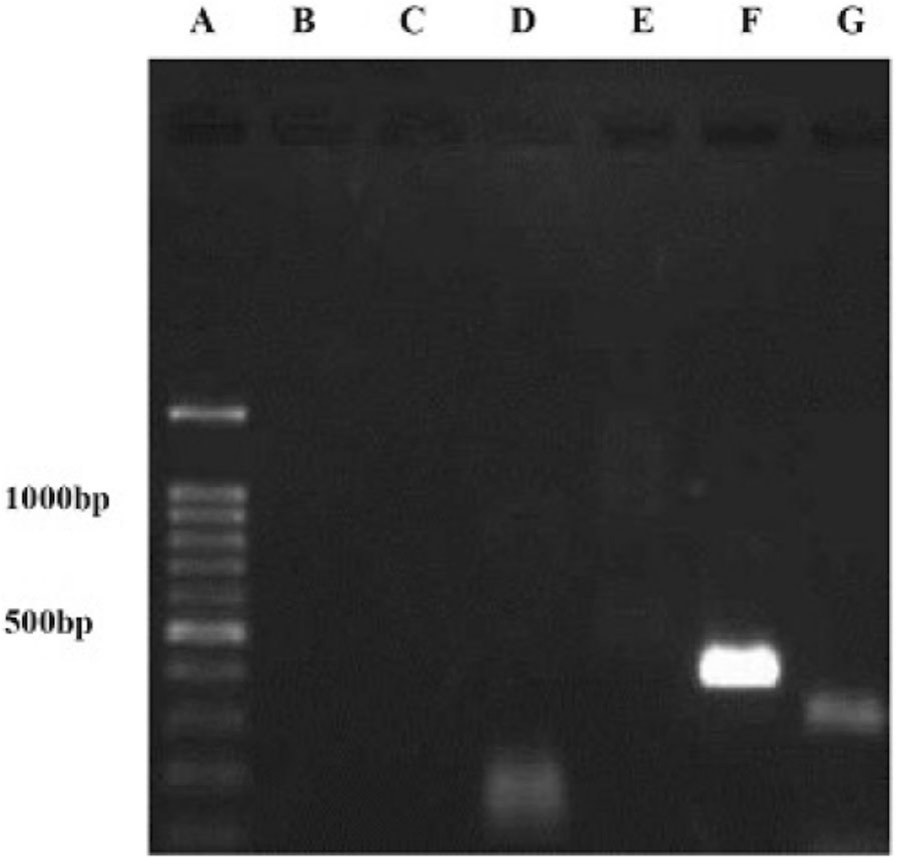
Amplification of Colicin *ANS4*, *E1*, *Iab* genes. A) DNA Ladder, B) Negative control, C) Negative control, D) Positive isolate for Colicin *ANS4* gene, E) Negative isolate, F) Positive isolate for Colicin *E1* gene, and G) Positive isolate for Colicin *Iab* gene.

**Fig. 4:**
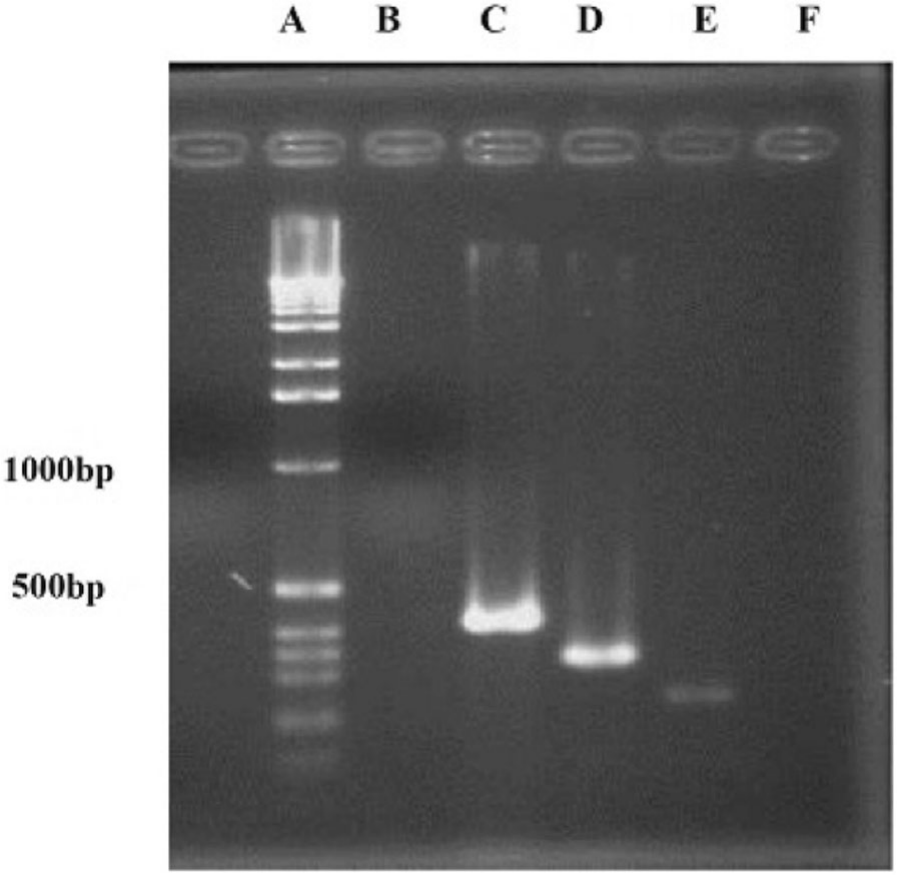
Detection of Colicin V, YU, and EMix genes. A) DNA Ladder, B) Negative control, C) Positive isolate for Colicin *V* gene, D) Positive isolate for Colicin *YU* gene, E) Positive isolate for Colicin *EMix* gene, and F) Negative control.

From one hundred and forty-six *E. coli* isolates of healthy sheep, eleven isolates (7.53%) contained *stx*_*1*_ and/or *stx*_*2*_ in combination with the *eae* or/and *ehly* genes and classified as STEC pathotype. Three isolates (2.05%) were positive for *cnf1* gene which categorized into NTEC pathotype (Fig. 1). One isolate (0.68%) was positive for *ipaH* gene that is an EIEC virulence gene and one hundred twenty *E. coli* isolates from healthy sheep did not have any virulence genes (Fig. 2). The frequency of bacteriocinogeny was 29.45% among healthy isolates and all types of colicin ending genes were detected in these isolates. The figures of colicin genes are shown in Figs. 3, 4.

Statistical analysis showed that the frequency of colicinogenic isolates in diarrhea associated *E. coli* was significantly higher than *E. coli* isolates from healthy sheep (*p* < 0.05). A specific association between ETEC and colicin V/ colicin Iab encoding genes in diarrheic *E. coli* isolates was observed (*p* < 0.05). In addition, a specific association between *ipaH* gene and Colicin V encoding gene was found (*p* < 0.05). Totally, *E. coli* isolates possessing no virulence determinant had (42/43, 97.67%) and (8/67, 11.94%) frequency of bacterocingeny in healthy and diarrheic isolates, respectively. Altogether, 110 colicin producer strains (37.67%) were identified of 292 *E. coli* isolates.

Approximately 28% of the 146 *E. coli* isolates from diarrheic sheep produced inhibitory activity against *E. coli* O157:H7 Sakai (EHEC). Five of these isolates (12.19%) possess more than one of the mentioned colicin target genes. The largest zone of inhibition belonged to col. E1 producer (12.32%) and col. V producer (10.27%) isolates. No zone of inhibition was obtained from diarrheic isolates which were positive for col. Iab and col. yu encoding genes. Thirty two (21.9%) healthy isolates had the inhibitory effect on *E. coli* O157:H7 Sakai (EHEC). All types of colicin in healthy isolates had the inhibitory effect on *E. coli* O157:H7 with the exception of col. Iab. Details of detected colicin encoding genes in relation to the different pathotypes and the inhibitory effect of the colininogenic isolates on *E. coli* O157:H7 Sakai (EHEC) are shown in Table 3.

**Table 3:** Relation between Colicin genes and pathotypes of *E. coli* isolates, and the inhibitory effect of the colininogenic isolates on *E. coli* O157:H7

Colicin genes	*Number of Isolates*		*Pathotypes*						Inhibition of *E. coli* O157:H7
			ETEC	aEPEC	STEC	NTEC	EIEC	Without Vir Genes	
*Colicin Iab*	*D*	25	22	0	0	0	0	3	–
	*H*	11	0	0	0	0	0	11	–
*Colicin E1*	*D*	18	1	1	14	2	0	0	+
	*H*	5	0	0	0	0	0	5	+
*Colicin V*	*D*	15	9	0	0	0	1	5	+
	*H*	5	0	0	0	0	1	4	+
*Colicin 5, 10, K*	*D*	3	0	2	1	0	0	0	+
	*H*	5	0	0	0	0	0	5	+
*Colicin YU*	*D*	1	0	0	1	0	0	0	–
	*H*	6	0	0	0	0	0	6	+
*Colicin EMix*	*D*	0	0	0	0	0	0	0	–
	*H*	3	0	0	0	0	0	3	+
*Colicin ANS4*	*D*	0	0	0	0	0	0	0	–
	*H*	2	0	0	0	0	0	2	+
*Multiple Colicin genes*	*D*	5	3	1	1	0	0	0	+
	*H*	6	0	0	0	0	0	6	+
*Total*	*D*	67	35 (52.23)	4 (5.97)	17 (25.37)	2 (2.98)	1 (1.49)	8 (11.9)	
	*H*	43	0	0	0	0	1 (2.32)	42 (97.6)	

Abbreviation: In this table: *ETEC* Entrotoxigenic E. coli, *a EPEC* atypical Entropathogenic E. coli, *STEC* Shiga Toxin producing E. coli, *NTEC* Necrotoxic E. coli, *EIEC* Entroinvasive E. coli; *D*: Diarrheic, *H* Healthy

Phylogenetic analysis of diarrheic and healthy isolates showed that the highest frequency of colicin encoding genes were found in B1 phylogroup (65.45%) followed by A (26.36%), D (7.27%) and B2 (0.9%) phylogroups. In B1 phylogroup, colicin E1 (17/45, 37.7%) and colicin Iab (11/27, 40.74%) encoding genes were more common in diarrheic and healthy *E. coli* isolates, respectively. Producer of colicin V belonged more frequently to A phylogroup in diarrheic and healthy *E. coli* isolates (22.38, 7.46%, respectively; *p* < 0.0001) when compared to other colicin producer isolates (Table 4).

**Table 4:** Detected Colicin genes in relation to phylogenetic groups/subgroups in *E. coli* isolated form diarrheic and healthy sheep

Target gene	Phylogenetic group		A		B1	B2		D	
	Phylogenetic subgroup		A0	A1	B1	B2–2	B2–3	D1	D2
*Colicin Iab*	D	25	–	–	20	–	–	5	–
	H	11	–	–	11	–	–	–	–
*Colicin E1*	D	18	1	–	17	–	–	–	–
	H	5	–	–	2	–	–	3	–
*Colicin V*	D	15	10	5	–	–	–	–	–
	H	5	4	1	–	–	–	–	–
*Colicin 5, 10, K*	D	3	–	–	3	–	–	–	–
	H	5	1	4	–	–	–	–	–
*Colicin Y, U*	D	1	–	–	–	1	–	–	–
	H	6	–	1	5	–	–	–	–
*Colicin EMix*	D	0	–	–	–	–	–	–	–
	H	3	–	–	3	–	–	–	–
*Colicin A, N, S4*	D	0	–	–	–	–	–	–	–
	H	2	–	2	–	–	–	–	–
*Multiple Colicin genes*	D	5	–	–	5	–	–	–	–
	H	6	–	–	6	–	–	–	–
*Total*	D	67	11	5	45	1	–	5	–
	H	43	5	8	27	–	–	3	–

## Discussion

In this study, the average prevalence of colicinogenic *E. coli* isolates from sheep was 37.67%. Surprisingly our data lies well within the recently published results from a different source for probiotic *E. coli* strains, humans [11, 14, 17, 18]. In those studies, Gordon et al., [19] detected 19 types bacteriocin genes when screening 266 *E. coli* strains that had been isolated from human fecal (38% of these isolates were bacteriocinogenic), and Smaj et al., [20] detected 29 bacteriocin types in 441 human fecal *E. coli* isolates (55% of which were bacteriocin-encoding isolates). Based on our presented data and the previously published results, sheep could also be considered as a potential source of the probiotic *E. coli* strains.

The results presented here showed that the frequency of bacteriocinogeny in *E. coli* isolates positively correlates with the number of detected virulence factors. To the best of our knowledge, this is the first time that the correlation between frequency of colicin encoding genes and the number of virulence factors in *E. coli* isolates from sheep have been investigated.

This study also showed that the prevalence of colicin E1 genes among diarrheic isolates was relatively high (10.27%) in comparison to the other colicin genes. Micenkova et al., [20] reported that colicin E1, which is a pore-forming bacteriocin, had a high prevalence in fecal and also in human UPEC isolates.

The prevalence of colicing Iab genes of *E. coli* isolates from diarrheic and healthy sheep (17.12 and 25.56%, respectively) were significantly higher than other colicin genes. This is in accordance with the study of Micenková., et al., [11, 14]. Another study showed that colicin Ia/Ib was one of the most frequent colicin types among ETEC isolates and also 38.2% of *Shigella sonnei* isolates produced colicin Ia/Ib [17]. In the present study, 15.06% of 292 of fecal isolates were positive for colicin Ia/Ib encoding gene and also categorized in ETEC pathotype.

The prevalence of colicin V encoding gene in healthy and diarrheic isolates was equal and there was a specific association between *ipaH* virulence gene and this type of colicin. This finding is in accordance with the previous studies, which showed that colicin V encoding plasmids are more commonly carried by the virulent *E. coli* isolates [7].

Furthermore, colicin Y is related to the colicin U that was isolated around 20 years ago in Europe from *Shigella boydii*. Colicin Y is rarely detected colicin type in Europe, but high frequency of colicin Y producers were detected in *E. coli* isolated in Amazonia [11]. In our study the prevalence of colicin Y/U genes was relatively low in both healthy and diarrheic isolates. Moreover, productions of nucelase colicins E2-E9 are also very rare in *E. coli* isolates. [18, 19, 21]. In this study, none of the diarrheic isolates were positive for colicin E2-E9 encoding genes.

Association of *E. coli* phylogroups and bacteriocin types has been tested in several previous publications, which produced different results. Gordon and O’Brien [19] tested a set of 266 fecal *E. coli* isolates and failed to find remarkable differences in the prevalence of bacteriocin determinants among the phylogroups. They reported phylogroup A and B1 tended to encode more colicin types, while in phylogroup A, genes encoding E1, Ia and V, were most common [21]. In this study, phylogenetic analysis of diarrheic and healthy isolates showed that the highest frequency of colicin encoding genes were found in B1 phylogroup (65.45%) followed by A (26.36%), D (7.27%) and B2 (0.9%) phylogroups. In phylogroup B1, colicin E1 and colicin Iab encoding genes were more common.

Setia et al., [16] showed that the colicin produced by 26.8% of *E. coli* isolates without known toxin genes recovered from healthy cattle were able to inhibit the growth of pathogenic *E. coli* K88. The present study showed that fifty *E. coli* isolates (50/292, 17.12%) from diarrheic and healthy sheep were able to produce different types of colicin, while they didn’t have any virulence factors. Thirty six isolates of them (12.32%) were able to inhibit the growth of *E. coli* O157:H7 Sakai (EHEC) in vitro. So, colicin produced by these isolates may potentially provide an effective strategy to reduce *E. coli* O157:H7 in food animals. In the recent study, all types of colicin were able to inhibit *E. coli* O157:H7 except for colicin Ia/Ib which is in agreement with the previous studies [22, 23]. Bradley et al.*,* [24] considered that O157 isolates were most likely resistant due to masking of the colicin Ia/Ib receptor by O-antigen side-chains of LPS. Another explanation for this resistance is that some *E. coli* O157:H7 isolates are colicinogenic and produce specific concomitant immunity proteins [23], they can be resistant to certain colicins or even a broad classes of colicins. [25]. On the other hand, this possibility should be considered that O157:H7 Sakai (EHEC) of human could have resistance against the colicins of strains isolated from sheep. Therefore, simultaneous administration of a mixture of several classes of colicins should be considered as a treatment to reduce *E. coli* O157:H7 (and other EHEC) in the gastrointestinal tract of food animals. [23]

In this study, all types of colicin encoding genes in *E. coli* isolated from healthy sheep were detected. Whereas, *E. coli* isolates from diarrheic sheep didn’t harbored colicin Mix E/ANS4 encoding genes. Previous studies found colicinogenic isolates from all sources, but the greatest types of colicin were from cats and sheep [9, 26]. Tahamtan et al., [27] reported that Ia/Ib and ANS4 colicin encoding genes had the highest frequency among *E.coli* strains isolates from healthy and diarrheic cattle in Shiraz, Iran. It can be concluded that many factors may influence the types of detected colicin genes including species, illness, diet and geographical conditions [28].

## Conclusion

The results and data presented in our report showed that the healthy sheep could be considered as a source of colicinogenic *E. coli* strains. These *E. coli* strains that do not harbor any virulent factors but are carrying the colicin genes are a potentially valuable source for anti-O175:H7 bacterial isolates. Further work is granted to study the in vivo inhibitory effects of these colicinogenic isolates of *E. coli* on the biocontrol of pathogenic O175:H7. On the other hand, it can be concluded that there is a correlation between presence of colicin genes and virulence genes in *E. coli* isolates which could be determined in the future studies.

## Abbreviation

a EPEC: atypical Entropathogenic *E. coli*
*chuA*: *E. coli* haem-utilization gene
*cnf*: cytotoxic necrotizing factor
*eae*: attaching and effacing gene of *E. coli*
EHEC: Enterohemorrhagic *E. coli*
EIEC: Entroinvasive *E. coli*
ETEC: Entrotoxigenic *E. coli*
HUS: Hemolytic Uremic Syndrome
*ipaH*: invasion plasmid antigen
LT: heat labile toxin.
NTEC: Necrotoxic *E. coli*
PCR: Polymerase Chain Reaction
SPSS: Statistical Package for the Social Sciences
ST: heat-stable enterotoxin
STEC: Shiga Toxin producing *E. coli*
*Stx*: Shiga toxin
TspE4.C2: an anonymous DNA fragment in *E. coli*
*yjaA*: *E. coli* K12 gene
